# Pneumopathie à éosinophile révélant un lymphome non hodgkinien de type B

**DOI:** 10.11604/pamj.2016.24.292.9138

**Published:** 2016-08-03

**Authors:** Siham Fikal, Hafsa Sajiai, Hind Serhane, Salma Aitbatahar, Lamyae Amro

**Affiliations:** 1 Service de Pneumologie, Hôpital Arrazi, CHU Mohammed VI, FMPM, Labo PCIM, UCA, Marrakech, Maroc

**Keywords:** Pneumonie, éosinophile, lymphome, Pneumonia, eosinophile, lymphoma

## Abstract

Le diagnostic de pneumonie à éosinophile est rare et l'étiologie maligne reste exceptionnelle. Les étiologies sont variables et sont dominées essentiellement par les affections allergiques et les causes médicamenteuses. Nous rapportons le cas d'un lymphome non hodgkinien de type B révélé par une pneumonie à éosinophile chez un patient de 61 ans. Le diagnostic de pneumonie à éosinophile a été confirmé par un taux d'éosinophile à 56% au lavage bronchoalvéolaire. L'étude immunohistochimique de la biopsie ostéomédullaire a révélé un lymphome malin non hodgkinien à petites cellules de phénotype B.

## Abstract (en)

The diagnosis of eosinophilic pneumonia is rare and malignant etiology remains exceptional. Eosinophilic pneumonia etiology varies and is mainly dominated by allergic and drug causes. We report the case of a 61-year-old patient with B-cell non-Hodgkin lymphoma revealed by eosinophilic pneumonia. The diagnosis of eosinophilic pneumonia was confirmed by eosinophil count of 56% in bronchoalveolar lavage. Immunohistochemical examination of bone marrow biopsy revealed malignant Small B cells non-Hodgkin lymphoma.

## Introduction

Le poumon à éosinophile est un ensemble d'affections caractérisées par des infiltrats pulmonaires, associés à une hyperéosinophilie sanguine ou alvéolaire, la prédominance des causes allergiques et parasitaires ne doit pas faire oublier la possibilité rare des causes néoplasiques dont les lymphomes non hodgkinien de type B. Les cellules malignes peuvent produire des cytokines actives dans la production d'éosinophiles, ce qui pourrait être responsable d'une éosinophilie avec possibilité de pneumonie à éosinophile.

## Patient et observation

**Figure 1:**
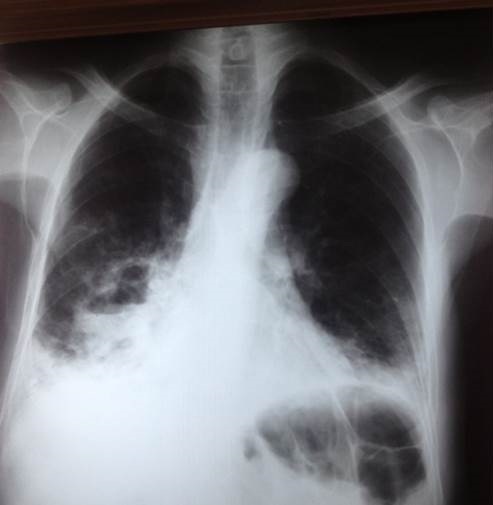
Radiographie thoracique de face objectivant deux opacités de type alvéolaires basithoraciques bilatérales excavés

**Figure 2:**
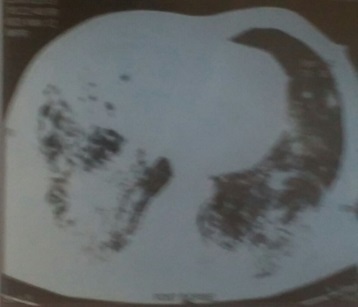
TDM thoracique montrant des infiltrats de type alvéolaires excavés

Nous rapportons l'observation d'un patient de 61 ans, non tabagique, diabétique type II sous insuline depuis 15 ans. Il présentait depuis 3 mois une toux productive ramenant des expectorations verdâtres, une douleur basithoracique bilatérale en point de côté et une dyspnée d'effort stade III de Sadoul, sans hémoptysie et sans autres signes extra-thoraciques évoluant dans un contexte d'asthénie et de fléchissement de l'état général. L'examen clinique a révélé un syndrome de condensation pulmonaire basal bilatéral avec une température à 37.5°. La radiographie thoracique a montré deux opacités de type alvéolaire basithoraciques bilatérales (**Figure 1**). La TDM thoracique a mis en évidence une atteinte bronchiolo-alvéolaire bilatérale (**Figure 2**). Le patient a été mis sous antibiothérapie à large spectre sans amélioration clinique ni radiologique. Un bilan a été réalisé: Le bilan biologique a révélé une hyperleucocytose à 18.000 elmts/mm^3^ avec une hyperéosinophilie à 780 elmts/mm^3^ sur la numération formule sanguine et un taux de LDH à 269 UI/L. Au bilan bactériologique, Les recherches de BK dans les expectorations étaient négatives à l'examen direct et en culture. Le bilan immunologique a montré une augmentation des alpha 2 globulines et des gammaglobulines sur l'électrophorèse des protéines sériques, un complément d'immunoélectrophorèse des protéines sériques a objectivé la présence d'immunoglobulines monoclonales de type IgM lambda. L'exploration endoscopique a trouvé un aspect inflammatoire diffus avec une hyperéosinophilie à 56% sur le lavage bronchoalvéolaire. Le diagnostic d'une hémopathie maligne était le diagnostic le plus probable. Le bilan a été complété par un myélogramme qui a objectivé une hyperplasie de la lignée mégacaryocytaire et absence d'infiltration plasmocytaire ou lympho-plasmocytaire, avec un phénotype kappa en faveur d'une prolifération B. Une biopsie ostéomédullaire a révélé un syndrome myéloprolifératif évoquant une lésion lymphomateuse dont le profil immunohistochimique est en faveur d'une localisation médullaire d'un lymphome malin non hodgkinien à petites cellules de phénotype B. La TDM abdomino-pelvienne n'a pas révélé d'anomalie, les sérologies VIH et EBV étaient négatives. Au terme de ce bilan, Le lymphome était classé IVB. Le patient était proposé pour chimiothérapie.

## Discussion

Le terme de syndrome hyperéosinophilique est utilisé chez les patients ayant une hyperéosinophilie sanguine ou tissulaire de toute cause (réactive, néoplasique, ou idiopathique) avec une preuve d'association d'une hyperéosinophilie et une atteinte tissulaire [**1**]. Il y'a trois sous-types de syndrome hyperéosinophilique (HES): variante myéloproliférative (HES- M), variante lymphocytaire (HES- L), et des HES d'étiologie inconnue [**2**]. La HES-L peut être le résultat d'une dérégulation de l'homéostasie des lymphocytes et généralement associée à une prolifération clonale des lymphocytes T de type 2 (Th2), ce qui entraîne une augmentation de la sécrétion de l'interleukine 5 (IL-5) [**3**]. Les lymphomes Lymphoblastiques sont principalement liés à la HES-M, tandis que les HES-L peuvent être associés aux lymphomes cutanés ou aux lymphomes périphériques à cellules T [**4**]. La survenue d'un lymphome systémique pourrait suggérer que la HES-L, associée à un phénotype aberrant des cellules T monoclonal, peut être un néoplasme à cellules T de bas grade ou un état précédant le lymphome. La discrimination entre les HES-L et le lymphome n'est pas facile car la prolifération peut posséder des caractéristiques communes de malignité des cellules T tels que le réarrangement clonal [**4**]. La plupart des patients atteints de pneumopathie chronique à éosinophile ont une hyperéosinophilie sanguine ce qui est le cas de notre patient. Celle-ci est une anomalie fréquente dans la pratique courante; ainsi que les pneumopathies à éosinophile qui sont le plus souvent de nature secondaire [**5**]. Les anomalies tomodensitométriques des pneumonies à éosinophiles décrites dans la littérature peuvent inclure les zones diffuses de verre dépoli, infiltrats alvéolaires, nodules mal définies et épaississement des septas interlobulaires, des anomalies qui ont été décrite sur la TDM de notre patient. Ce diagnostic est confirmé par les résultats de cytologie du lavage broncho-alvéolaire qui montre un taux élevé d'éosinophiles > 30%, ce qui était le cas de notre patient [**6**]. Kawazaki et al ont décrit le cas d'un lymphome à cellule T chez une femme de 57 ans, associé à une pneumonie à éosinophile avec une pleurésie à éosinophile [**7**]. Jacobs et al ont rapporté le cas d'un lymphome lymphocytaire chez un homme de 32 ans, confirmé par biopsie ganglionnaire, associé à une hyperéosinophilie et fibrose endomyocardique [**8**]. Cependant, Kim et al ont rapporté un cas de syndrome d'hyperéosinophilie idiopathique évoluant vers un lymphome à cellules T chez une fillette de 3 ans [**9**]. Ces cas rapportés restent sporadiques et très rares, d'où l'intérêt de notre observation. Le pronostic des lymphomes associés à une hyperéosinophilie est péjoratif à la fois chez les enfants et les adultes, avec une survie médiane de 7,5 mois [**10**].

## Conclusion

Nous avons rapporté un cas exceptionnelde lymphome malin non hodgkinien révélé par une pneumopathie à éosinophile. Cette association rare est de pronostic péjoratif par rapport aux lymphomes malins non hodgkiniens isolés. D'où l'intérêt de penser à l'origine lymphomateuse devant toute pneumopathie à éosinophile, pour un diagnostic précoce et une meilleure prise en charge.
